# Pitpnm1 is expressed in hair cells during development but is not required for hearing^☆^

**DOI:** 10.1016/j.neuroscience.2013.06.045

**Published:** 2013-09-17

**Authors:** F.A. Carlisle, S. Pearson, K.P. Steel, M.A. Lewis

**Affiliations:** Wellcome Trust Sanger Institute, Wellcome Trust Genome Campus, Hinxton, Cambridge, Cambs CB10 1SA, United Kingdom

**Keywords:** ABR, auditory brain stem response, ANOVA, analysis of variance, E, embryonic day, EDTA, ethylenediaminetetraacetic acid, P, postnatal day, PCR, polymerase chain reaction, Pitpnm1, inner ear, hair cell, mouse mutant, deafness, Mir96

## Abstract

Deafness is a genetically complex disorder with many contributing genes still unknown. Here we describe the expression of Pitpnm1 in the inner ear. It is expressed in the inner hair cells of the organ of Corti from late embryonic stages until adulthood, and transiently in the outer hair cells during early postnatal stages. Despite this specific expression, *Pitpnm1* null mice showed no hearing defects, possibly due to redundancy with the paralogous genes *Pitpnm2* and *Pitpnm3*.

## Highlights

• We studied the expression of Pitpnm1 in the developing mouse inner ear.
• We covered several ages between E14.5 and P5, and also looked at adults.
• Pitpnm1 is expressed in the inner hair cells from before birth to adulthood.
• Pitpnm1 is expressed transiently in the outer hair cells at early postnatal stages.
• Mice lacking Pitpnm1 display no obvious auditory defects.

## Introduction

Deafness is the most common sensorineural disorder in the human population and is a particular problem in the older population; 60% of people over the age of 70 have a hearing loss of 25 dB or more, and would benefit from a hearing aid (Davis, 1995). Many genes are known to cause deafness in humans, but there are many more loci for which the gene responsible remains unknown (http://hereditaryhearingloss.org). One way to discover new genes involved in hearing is to carry out an expression screen, looking for genes specifically expressed in the inner ear. However, expression of a gene in a specific organ does not mean that the gene is critical for the development or function of that organ; studies of lack-of-function mutants are a necessary complement to an interesting expression pattern. Here we present a functional study of and expression data for such a gene which was found to be specifically expressed in hair cells.

*Pitpnm1*, also known as *Nir2* or *RdgB*, was first found to be expressed in the hair cells in a study of mice mutant for the microRNA *Mir96*, being one of several genes that were markedly downregulated in the mutant (Lewis et al., 2009). The gene encodes a phosphatidylinositol transfer protein bound to the cytosolic side of the Golgi apparatus membrane (Aikawa et al., 1997, 1999; Lev et al., 1999; Litvak et al., 2002a). It is a homologue of the *Drosophila retinal degeneration B* gene, as are the two other members of the same family, *Pitpnm2* and *Pitpnm3* (Aikawa et al., 1997; Lev et al., 1999; Lu et al., 1999). Flies lacking *RdgB* display rapid photoreceptor degeneration (Harris and Stark, 1977).

The Pitpnm1 protein has two major functions: to regulate diacylglycerol (DAG) levels in the Golgi apparatus membrane and to regulate RhoA function (Litvak et al., 2002b, 2005; Tian et al., 2002). Through its phosphatidylinositol transfer domain, Pitpnm1 upregulates DAG and thereby increases the recruitment of other proteins required for *trans* Golgi network to plasma membrane transport (Jamora et al., 1999; Baron and Malhotra, 2002; Litvak et al., 2005). Pitpnm1 also helps maintain the Golgi apparatus structure (Litvak et al., 2005). With regard to RhoA, Pitpnm1 has an amino-terminal Rho inhibitory domain, which allows it to bind GDP–RhoA and prevent it from exchanging GDP (Guanosine diphosphate) for GTP Guanosine triphosphate (Tian et al., 2002). This inhibition of RhoA promotes cell extension, and facilitates cytokinesis since RhoA activity prevents cell division (Etienne-Manneville and Hall, 2002; Litvak et al., 2002b, 2004; Tian et al., 2002; Servotte et al., 2006; Morin et al., 2009).

miR-96 is a master regulator of inner ear development and is thought to coordinate a network of genes (Kuhn et al., 2011); thus, *Pitpnm1* was an excellent candidate for further study of its role in inner ear function. We therefore examined its expression at different time points during development and characterised the hearing of mice lacking the gene.

## Experimental procedures

### Sample collection and preparation

Wildtype mice from the C57BL/6J-Tyr^c-Brd^ strain were used for the main expression study. Embryonic samples were collected at embryonic day (E) 14.5, E16.5, and E18.5; the day of plug discovery was deemed E0.5. Postnatal pups were collected at the day of birth (P0), P3, P4 (for the *Pitpnm1* null mice) and P5. The 9-week-old mice were from a mixed background of C57BL/6J-Tyr^c-Brd^ and C57BL/6N. The heads for all samples were bisected, fixed in 10% formalin for two days, dehydrated and embedded in paraffin wax. The half heads from the 9-week-old mice were decalcified for two weeks in 10% EDTA after washing and before dehydration.

### Immunohistochemistry

Embedded samples were cut into 8 μm thick sections along the sagittal plane. Immunohistochemistry was then carried out on slides using the Ventana Discovery machines (Ventana Roche, Tucson, AZ, USA) with the manufacturer’s reagents (CC1 (cat. no. 950-124), EZPrep (cat. no. 950-100), LCS (cat. no. 650-010), RiboWash (cat. no. 760-105), Reaction Buffer (cat. no. 95-300), and RiboCC (cat. no. 760-107)) according to the manufacturer’s instructions. The DABMap™ Kit (Ventana; cat. no. 760-124) with haematoxylin counterstain (cat. no. 760-2021) and bluing reagent (cat. no. 760-2037) was used. Slides covering the entire inner ear for three different mice at each age were stained, and the observed expression patterns were only considered reliable if present in all three samples. The primary antibody used was Abcam (Cambridge, Cambs, UK) goat polyclonal antibody to Nir2 (Pitpnm1) (ab22823), with the Jackson ImmunoResearch (West Grove, PA, USA) biotin-conjugated donkey anti-goat (705-065-147) secondary. All antibodies were diluted in ‘Antibody staining solution’: 10% foetal calf serum, 0.1% Triton, 2% bovine serum albumin (BSA) and 0.5% sodium azide in phosphate-buffered saline (PBS).

Controls were run for the secondary antibody, wherein the above immunohistochemistry protocol was used on slides at each age but the primary antibody was replaced with an equal volume of antibody staining solution. Some staining was observed in certain patches of brain tissue, but none at all in the cochlea or vestibular system.

### Analysis

Stained slides were examined and images obtained using an AxioCam HRc camera mounted on a Zeiss microscope. Images were then processed in Photoshop CS2.

### Generation and testing of knockout mice

The *Pitpnm1* mutant allele (*Pitpnm1^tm1a(EUCOMM)Wtsi^*) is a “knockout-first” allele generated by the Sanger Institute Mouse Genetics Project as described in (Skarnes et al., 2011). The mutant was maintained on a C57BL/6N background. P4 pups were collected as described in Section “Sample collection and preparation” and tested with the same antibody used for the expression study. Genotyping was carried out by polymerase chain reaction (PCR) with the following primers: Wt_F: TCCCCTGAGGTAGTTGCTGAC; Wt_R: ATGTTGGCCCTGTGATGTTG; Mutant_F: TCCCCTGAGGTAGTTGCTGAC; Mutant_R: TCGTGGTATCGTTATGCGCC. The wildtype PCR gives a band of 355 bp in wildtypes and heterozygotes and the mutant PCR a band of 128 bp in heterozygotes and homozygotes.

### Auditory brainstem response

In order to assess the hearing of mice homozygous for the *Pitpnm1* mutation, mice were tested using auditory brainstem response measurements (ABR). Mice homozygous, heterozygous or wildtype for the targeted mutation *Pitpnm1^−/−^*, were anaesthetised and ABR recordings made by placing subcutaneous hook electrodes through the skin. Pure tone pips were presented in 5 ms bursts with a 1 ms rise/fall time, and responses to 6, 12, 18, 24 and 30 kHz were recorded, as were responses to multi-frequency 0.01 ms clicks. For some mice, additional frequencies were tested (3, 36 and 42 kHz). The responses to 256 repetitions were averaged using custom software, and the thresholds determined by identifying the lowest sound intensity at which any part of the ABR waveform was visually discernable (Ingham et al., 2011). Three ages were studied; 7, 14 and 24 weeks, and at least seven mice were tested for each genotype and age. A two-way analysis of variance (ANOVA) was used to determine whether there were significant differences between the genotypes at each frequency.

### Real time PCR (RT-PCR) on *Pitpnm* genes

The organs of Corti of three P5 C57BL/6J-Tyr^c-Brd^ mice were dissected out and stored at −20 °C in RNAlater stabilization reagent (cat. no. 76106, QIAGEN (Valencia, CA, USA)). RNA was extracted using QIAshredder columns (QIAgen, cat. no. 79654) and the RNeasy mini kit (QIAgen, cat. no. 74104), then treated with DNAse 1 (cat. no. AMP-D1, Sigma (St Louis, MO, USA)), all according to the manufacturer’s instructions. cDNA was generated from normalised quantities of RNA using the Superscript II Reverse Transcriptase kit (cat. no. 11904-018, Invitrogen (Carlsbad, CA, USA)). PCR was run with a touchdown protocol. Primer sequences were designed to cover at least one exon–exon junction:

$$\begin{array}{ccc} \text{Pitpnm}1 & \text{F:TACCCATATACCCGGACACG} & \text{R:CTCTTCCGCTTTGTATTCTCC}\\ \text{Pitpnm}2 & \text{F:GAGTCCTGGAATGCCTACCC} & \text{R:GACTGACTGGAACAGCTTGG}\\ \text{Pitpnm}3 & \text{F:TGCTCGGAGGCTTTCTCG} & \text{R:AGGGTCCCGCACTGTAGC} \end{array}$$

All experiments were performed in accordance with the UK Home Office Animals (Scientific Procedures) Act 1986.

## Results

### Expression in wildtypes

**Fig. 1:**
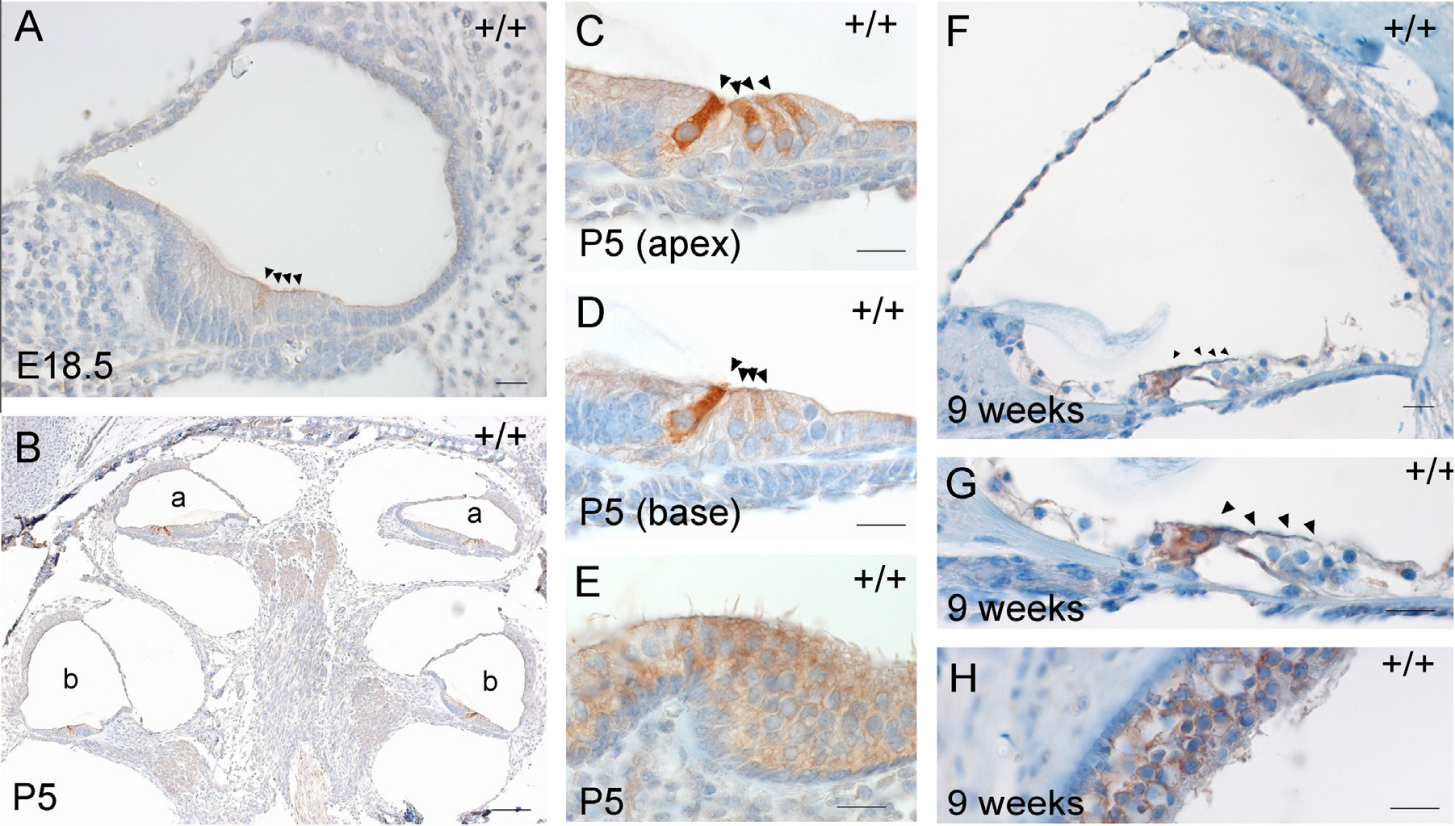
Expression of *Pitpnm1*. Pitpnm1 expression in wildtype mice at E18.5 (A), P5 (B–E) and 9 weeks old (F–H). (A) Only inner hair cells show expression at E18.5. (B) The organ of Corti at P5, with staining in all hair cells at the apex (a) but in the inner hair cell only at the base (b). (C) and (D) are close-ups of the apical turn and basal turn respectively. (E) Fainter staining in vestibular hair cells and supporting cells of the crista at P5. (F) Staining in the inner hair cells only at 9 weeks old; (G) is a close-up of (F). (H) Staining in the macula at 9 weeks old. Positive staining is in brown; tissue is counterstained blue. Cochlear hair cells are indicated by arrowheads. (A, C–H) Scale bar = 20 μm, (B) Scale bar = 100 μm.

Specific expression of *Pitpnm1* was visible from E18.5, when inner hair cells showed faint staining (Fig. 1A). Expression at P0 was generally identical to that seen at E18.5 but was more clear and well established (data not shown). By P3, all hair cells showed strong expression of *Pitpnm1* (data not shown). Strikingly, at P5 all inner hair cells still maintained heavy expression, but outer hair cells showed a steep gradient: at the base outer hair cells showed essentially no *Pitpnm1* expression, while at the apex expression in outer hair cells was similar to P3 levels (Fig. 1B–D). In the vestibular system, moderate levels of *Pitpnm1* expression could be seen in both hair cells and supporting cells from E18.5, and were maintained at a fairly constant level up to P5 (Fig. 1E). At 9 weeks old, Pitpnm1 staining was still faintly visible in the inner hair cells and the vestibular system (Fig. 1F–H).

### *Pitpnm1^−/−^* mouse phenotyping

**Fig. 2:**
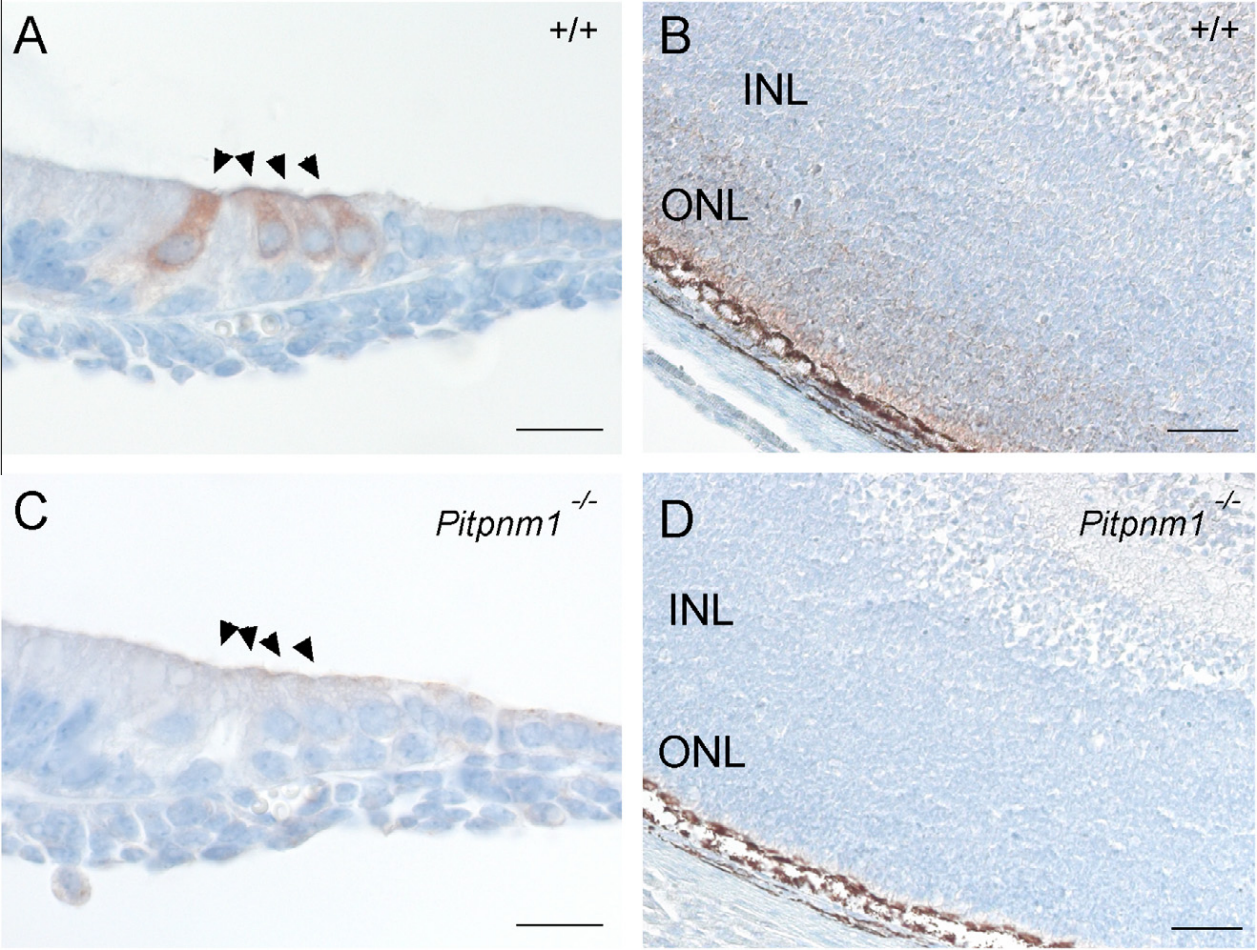
Pitpnm1 expression in homozygote knockout mice at P4. Pitpnm1 expression is present at P4 in wildtype mice (A, B) but not in a homozygote littermate (C, D), in both hair cells (A, C; the middle turn of the cochlea is shown) and the retina (B, D). Positive staining is indicated by brown; tissue is counterstained blue (the brown cells in D are melanocytes in the retinal pigment epithelium). Hair cells are indicated by arrowheads. INL = inner nuclear layer; ONL = outer nuclear layer. Scale bar in A and C = 20 μm, bar in B and D = 50 μm.

*Pitpnm1* knockout mice are viable and fertile. The absence of Pitpnm1 protein in the knockout animals was confirmed by immunohistochemistry; *Pitpnm1^−/−^* animals had no expression in the hair cells or the retina (Fig. 2). The homozygous *Pitpnm1* mutants were subjected to a broad phenotypic screen through the Sanger Institute Mouse Genetic Programme (http://www.sanger.ac.uk/mouseportal/). The only tests where *Pitpnm1* null mice show a difference from the wildtype are the plasma chemistry tests, where male homozygotes showed a decreased circulating cholesterol level and hypocalcaemia (http://www.sanger.ac.uk/mouseportal/phenotyping/MCTJ/plasma-chemistry/), and the haematology test, where female homozygotes show a decrease in leucocyte cell number (http://www.sanger.ac.uk/mouseportal/phenotyping/MCTJ/haematology-cbc/). No obvious histopathological defects were observed in the eyes, and no consistent eye phenotype was visible in 13 mutants examined using a slit lamp, ophthalmoscope and endoscope (data not shown; http://www.sanger.ac.uk/mouseportal/, http://www.sanger.ac.uk/mouseportal/phenotyping/MCTJ/eye-morphology/).

**Fig. 3:**
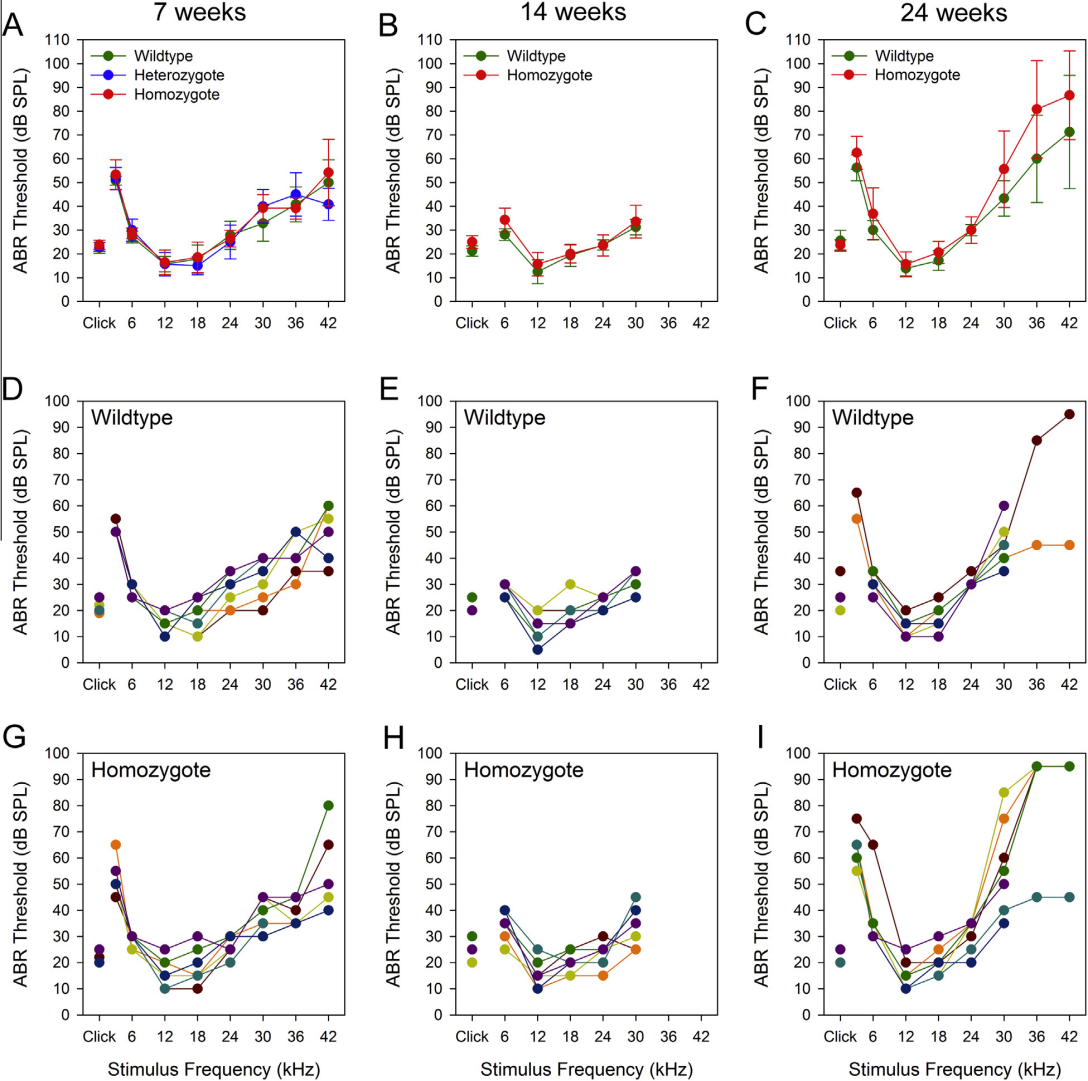
ABR results of *Pitpnm1* null mice. (A–C) ABR results showing average hearing thresholds from 3 to 42 kHz for wildtype (green), heterozygous (blue) and homozygous (red) mutant *Pitpnm1* mice at 7 weeks (A), 14 weeks (B) and 24 weeks (C). Thresholds for click stimuli are shown as a single data point on the left of the graph. No difference is visible between wildtypes and mice carrying the null allele at 7 weeks (ANOVA, *p* = 0.322), and although there is a significant difference in 14-week-old mice at 6 kHz (ANOVA, *p* = 0.009), the difference is too small to be biologically meaningful. There is also a significant difference in 24-week-old mice at 30, 36 and 42 kHz (ANOVA, *p* = 0.022, 0.004 and 0.03 respectively), but in this case examination of the individual traces (F, I) show that the wildtypes are just as variable. Error bars are standard deviation. D–I) Thresholds for individual mice (one colour per mouse) at the three ages (D, G – 7 weeks; E, H – 14 weeks; F, I – 24 weeks), grouped by genotype; D–F are wildtypes and G–I homozygotes.

All *Pitpnm1^−/−^* homozygote animals had normal waveforms, wave amplitudes and latencies at all ages tested (data not shown). There was no significant difference in ABR threshold between wildtype and homozygote null animals at 7 weeks (ANOVA, *p* = 0.322, Fig. 3A). At 14 weeks there is a slight increase in threshold in homozygotes at 6 kHz (Fig. 3B) but it is not sufficient to be biologically meaningful. At 24 weeks, some individual mice showed increased thresholds at high frequencies, but this occurred in homozygotes and wildtypes and is probably just a reflection of variable age-related hearing loss on the C57BL/6N background (Fig. 3E, F, H, I).

### Expression of *Pitpnm1*, *Pitpnm2* and *Pitpnm3* in wildtypes

**Fig. 4:**
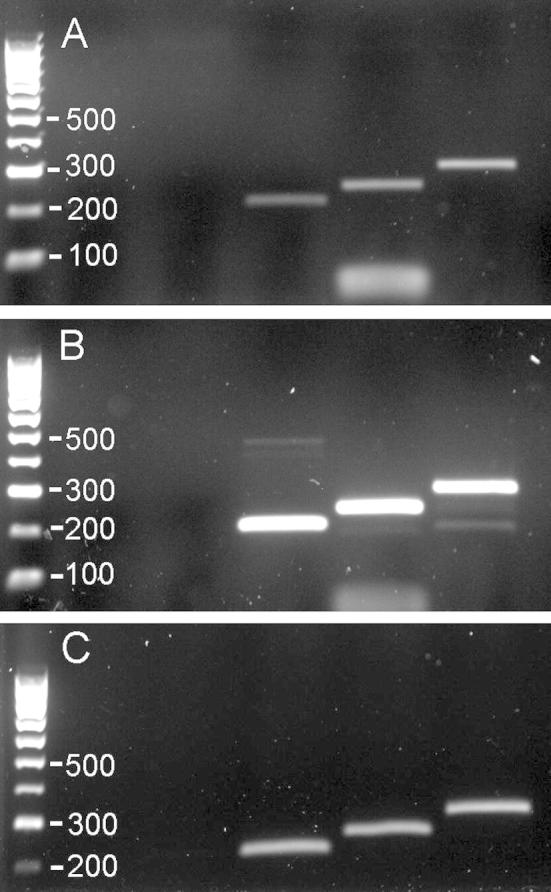
Organ of Corti RT-PCR for *Pitpnm* genes. RT-PCR results showing bands for *Pitpnm1* (left), *Pitpnm2* (centre) and *Pitpnm3* (right) from cDNA from the organ of Corti of three separate mice (A–C). Ladder band size is shown on each figure; the *Pitpnm* bands are between 200 and 350 bp in size.

cDNA made from RNA from the organ of Corti of P5 wildtype mice was used as the template for PCR with primers against the spliced mRNA sequences of *Pitpnm1*, *Pitpnm2* and *Pitpnm3*. All three genes produced a clear band in the samples tested, indicating that they are all expressed in the organ of Corti (Fig. 4).

## Discussion

The expression pattern of *Pitpnm1* in the inner ear is both time- and cell-specific. The most intense expression at any stage is always seen in the inner hair cell, starting at E18.5 and remaining present there in adult ears. It is interesting that constant expression of *Pitpnm1* at prenatal stages and in adults was seen in both inner hair cells and vestibular hair cells, while expression in the outer hair cells was transient, appearing to spread from the apex towards the base between P0 and P3, then retract from the base and become limited to the apex by P5. By 9 weeks old there was no expression detected in the outer hair cells. Both the inner and vestibular hair cells are responsible for transducing the sensory stimuli to afferent neural activity, while the outer hair cells, a feature specific to mammals, modulate the sensitivity of the basilar membrane. The difference in expression of Pitpnm1 may reflect these differences in function, specifically, the need for motility in outer hair cells. Pitpnm1 is known to inhibit RhoA, a GTPase important for the regulation of outer hair cell motility (Kalinec et al., 2000), and thus the absence of Pitpnm1 in mature outer hair cells may be a necessary feature specific to the outer hair cells to enable them to function. However, the role of Pitpnm1 in the vestibular and inner hair cells, a role which must either be unnecessary in outer hair cells or performed by another protein, remains unknown.

It is intriguing that despite the very specific expression in hair cells Pitpnm1 is not required for normal hearing. Although several of the older *Pitpnm1^−/−^* mice have raised thresholds, so do some of the wildtypes (Fig. 3F, I). The C57BL/6N strain is known to suffer from progressive deafness, and it is likely that this is causing the raised thresholds in some of the older animals tested. The absence of an auditory phenotype in mice lacking *Pitpnm1* implies that the reduced *Pitpnm1* expression seen in the *Mir96* mutant does not contribute to its phenotype (Lewis et al., 2009).

The homozygous null mice showed very little difference to their wildtype siblings across a wide range of tests (http://www.sanger.ac.uk/mouseportal/phenotyping/MCTJ). Where they do differ, the differences are sex-specific, and in the case of the decreased circulating cholesterol levels of the male homozygotes, the readings cover a wide range of values, from higher than the wildtypes to much lower, and the difference may not be biologically meaningful. However, the potential link to hypocalcaemia is interesting because Pitpnm1 is a calcium-binding protein (Lev et al., 1999).

The lack of detection of Pitpnm1 by immunohistochemistry in either eye or ear in homozygous mutants (Fig. 2) demonstrates that the knockout is efficient. However, there are three *Pitpnm* genes, all of which are homologous to the *Drosophila retinal degeneration B* gene, and all of which are expressed in the organ of Corti (Fig. 4), so it is possible that one or both of the others are compensating for the lack of *Pitpnm1*. A knockout of *Pitpnm2* has been studied and was found to have no phenotype despite specific expression in the retina (Lu et al., 2001), and it is notable that *Pitpnm1* is also expressed in the retina (Fig. 2B) but no eye or vision abnormalities were observed in the *Pitpnm1* knockout. No *Pitpnm3* knockouts have been reported. It would be interesting to study a double or triple knockout, and determine if the lack of a phenotype in *Pitpnm1* and *Pitpnm2* null mice is due to compensation by the other Pitpnm family members.

## Author contributions

The expression study was carried out by F.C. and ABR was done by S.P. M.L. did the staining of the mutant mouse ear and eye, and the RT-PCR on the three *Pitpnm* genes. The study was designed by K.P.S. and M.L., and the paper was written by F.C., M.L. and K.P.S.
